# Tumor necrosis factor α sensitizes spinal cord TRPV1 receptors to the endogenous agonist *N*-oleoyldopamine

**DOI:** 10.1186/1742-2094-7-49

**Published:** 2010-08-26

**Authors:** Diana Spicarova, Jiri Palecek

**Affiliations:** 1 Department of Functional Morphology, Institute of Physiology, Academy of Sciences of the Czech Republic, Prague, Czech Republic

## Abstract

Modulation of synaptic transmission in the spinal cord dorsal horn is thought to be involved in the development and maintenance of different pathological pain states. The proinflamatory cytokine, tumor necrosis factor α (TNFα), is an established pain modulator in both the peripheral and the central nervous system. Up-regulation of TNFα and its receptors (TNFR) in dorsal root ganglion (DRG) cells and in the spinal cord has been shown to play an important role in neuropathic and inflammatory pain conditions. Transient receptor potential vanilloid 1 (TRPV1) receptors are known as molecular integrators of nociceptive stimuli in the periphery, but their role on the spinal endings of nociceptive DRG neurons is unclear. The endogenous TRPV1 receptor agonist *N*-oleoyldopamine (OLDA) was shown previously to activate spinal TRPV1 receptors. In our experiments the possible influence of TNFα on presynaptic spinal cord TRPV1 receptor function was investigated. Using the patch-clamp technique, miniature excitatory postsynaptic currents (mEPSCs) were recorded in superficial dorsal horn neurons in acute slices after incubation with 60 nM TNFα. A population of dorsal horn neurons with capsaicin sensitive primary afferent input recorded after the TNFα pretreatment had a basal mEPSC frequency of 1.35 ± 0.20 Hz (n = 13), which was significantly higher when compared to a similar population of neurons in control slices (0.76 ± 0.08 Hz; n = 53; P < 0.01). In control slices application of a low concentration of OLDA (0.2 uM) did not evoke any change in mEPSC frequency. After incubation with TNFα, OLDA (0.2 uM) application to slices induced a significant increase in mEPSC frequency (155.5 ± 17.5%; P < 0.001; n = 10). Our results indicate that TNFα may have a significant impact on nociceptive signaling at the spinal cord level that could be mediated by increased responsiveness of presynaptic TRPV1 receptors to endogenous agonists. This could be of major importance, especially during pathological conditions, when increased levels of TNFα and TNFR are present in the spinal cord.

## Background

The cytokine, tumor necrosis factor α (TNFα), is now well established as a pain modulator in both the peripheral and central nervous systems [1]. There is now mounting evidence of TNFα involvement in inflammatory, neuropathic and cancer-related pain [2]. Several studies have shown a correlation between the level of TNFα expression and the development of allodynia or hyperalgesia [2-5]. Besides increased local TNFα synthesis and release during peripheral inflammation, TNFα up-regulation has also been demonstrated in dorsal root ganglion (DRG) neurons [6-8] and spinal cord [3,9,10] in experimental models of peripheral neuropathy, including chronic constriction injury (CCI), L5 spinal nerve transection or sciatic nerve crush. It has been suggested that during neuropathy or peripheral inflammation TNFα could be released in the spinal cord mainly from activated glial cells [9-11].

The effect of TNFα is mediated by two receptors: TNFR1 (p55) and TNFR2 (p75). Both receptors have been detected in DRG and spinal cord neurons [12,13]. In different peripheral neuropathy models, TNFR1/2 receptors are up-regulated in DRG neurons [14-16] and TNFR1 in the spinal cord dorsal horn (DH) [10]. Later studies localized TNFR2 expression exclusively in non-neuronal DRG cells after lipopolysaccharide (LPS) treatment [17] or after inflammation induced by complete Freund's adjuvant (CFA) [18]. It was recently demonstrated that TNFR2 receptors are crucial for the development of heat hyperalgesia in a cancer-related pain model in mice [4].

Nociceptive DRG neurons express transient receptor potential vanilloid 1 (TRPV1) receptors, which are localized on their peripheral and central endings [19]. In peripheral tissue they serve as molecular integrators of nociceptive stimuli. However, the function of spinal TRPV1 receptors is not fully understood. As temperature increases or pH decreases, which activate TRPV1 receptors in the periphery, do not occur in the spinal cord, great effort was needed to detect possible endogenous activators of central TRPV1 receptors [20]. Recently, several lipids have been described as potential endogenous agonists of TRPV1 receptors. Most of them also activate cannabinoid receptors, similar to anandamide (AEA, *N*-arachidonoylethanolamine), which was one of the first substances suggested to act as an endogenous TRPV1 receptor ligand [21]. AEA has been shown to excite cutaneous C nociceptors via TRPV1 receptors activation and to evoke nocifensive behaviour after peripheral application *in vivo *[22]. Intrathecal AEA administration has been demonstrated to have an analgesic effect, while higher concentrations also evoke pain-related behavior [23]. Other potential endogenous activators of TRPV1 receptors are products of lipoxygenases [24], omega-3 polyunsaturated fatty acids [25] or unsaturated *N*-acyldopamines originally isolated from bovine striatum as *N*-arachidonoyldopamine (NADA) [26]. Further analysis of striatal extracts resulted in the identification of, among other acyldopamines, *N*-oleoyldopamine (OLDA), which induces thermal hyperalgesia after intraplantar application and possesses a high potency of putative endovanilloid in mobilization of intracellular calcium in TRPV1-transfected cells [27]. Unlike NADA, OLDA is only a weak ligand for rat CB1 receptors; but is recognized by the anandamide membrane transporter while being a poor substrate for fatty-acid amide hydrolase (FAAH) [27]. Behavioral experiments have shown thermal hyperalgesia following intrathecal OLDA administration [28]. Our previous electrophysiological recordings in spinal cord slices demonstrated that application of 10 μM OLDA solution increases mEPSC frequency in superficial DH neurons due to specific TRPV1 receptor activation, as demonstrated by TRPV1 antagonists (SB366791, BCTC) application [28]. The concentration of OLDA needed to activate TRPV1 receptors decreased dramatically from 10 μM under control conditions to 0.2 μM after PKC activation, pretreatment with the inflammatory mediator bradykinin, or in a model of peripheral inflammation [28].

It has been shown previously that TRPV1 receptor expression and sensitivity to capsaicin may be modulated by TNFα pretreatment [4,29-31]. In our experiments we have studied modulation of spinal cord presynaptic TRPV1 receptor function by TNFα. We have recorded the frequency of mEPSCs from superficial dorsal horn neurons in spinal cord slices in order to investigate the effect of acute slice incubation with cytokine TNFα on TRPV1 receptor activation by the endogenous agonist, OLDA.

## Methods

Acute spinal cord slices were prepared from male Wistar rats on postnatal days P19 to P23, as was described previously [28]. After anesthesia with pentobarbital sodium (90 mg/kg), the lumbar spinal cord was removed and immersed in oxygenated ice-cold dissection solution containing (in mM): 95 NaCl, 1.8 KCl, 7 MgSO_4_, 0.5 CaCl_2, _1.2 KH_2_PO_4_, 26 NaHCO_3_, 25 D-glucose, 50 sucrose. The spinal cord was fixed to a vibratome stage using cyanoacrylate glue (Leica, VT 1000 S, Germany) in a groove between two agar blocks. Acute transverse slices 300 - 350 μm thick were cut, incubated in the dissection solution for 30 min at 33°C and then stored in a recording solution at room temperature and allowed to recover for 1 h before the electrophysiological experiments. Recording solution contained (in mM): 127 NaCl, 1.8 KCl, 1.2 KH_2_PO_4_, 2.4 CaCl_2, _1.3 MgSO_4_, 26 NaHCO_3_, 25 D-glucose. For the actual measurement, slices were transferred into a recording chamber that was perfused continuously with recording solution at a rate >2 ml/min. All extracellular solutions were saturated with carbogen (95% O_2_, 5% CO_2_) during the whole process.

Patch-clamp recordings were made from individual DH neurons visualized using a differential interference contrast (DIC) microscope (Leica, DM LFSA, Germany) equipped with an infrared-sensitive camera (IR camera Hitachi KP-200P, Japan) with a standard TV/video monitor (Hitachi VM-172, Japan). Patch pipettes were pulled from borosilicate glass tubing (Rückl Glass, Otvovice, Czech Republic). When filled with intracellular solution, they had resistances of 3.5 - 7.0 MΩ. The intracellular pipette solution contained (in mM): 125 gluconic acid lactone, 15 CsCl, 10 EGTA, 10 HEPES, 1 CaCl_2_, 2 Na_2_ATP, 0.5 NaGTP and was adjusted to pH 7.2 with CsOH. Voltage-clamp recordings in the whole-cell configuration were performed with an AxoPatch 1 D amplifier (Molecular Devices, USA) at room temperature (21°C - 24°C). Whole-cell responses were low-pass filtered at 2 kHz and digitally sampled at 10 kHz. The series resistance of neurons was routinely compensated by 80% and was monitored during the whole experiment. AMPA receptor-mediated mEPSCs were recorded from superficial DH neurons in laminae I and II, clamped at -70 mV in the presence of 10 μM bicuculline, 5 μM strychnine and 0.5 μM tetrodotoxin (TTX). The software package pCLAMP version 9.0 (Axon Instruments, Inc., Foster City, CA, USA) was used for data acquisition and subsequent off-line analysis. Neurons with capsaicin-sensitive primary afferent input were identified by the increase of mEPSC frequency (> 20%) following capsaicin (0.2 μM) administration at the end of the experimental protocol.

All drugs used in this study were of analytical grade and purchased from Sigma-Aldrich (Prague, Czech Republic) or Tocris Bioscience (Bristol, UK). TNFα was dissolved in 0.1% BSA; capsaicin and OLDA were dissolved in dimethylsulfoxide (DMSO), which had a concentration < 0.1% in the final solution.

Data segments of 2 min duration were analyzed for each experimental condition. Only mEPSCs with an amplitude of 5 pA or greater (which corresponded to at least twice the noise level) were included in the frequency analysis. In the case of amplitude analysis, the same events and data segments were used. Data are expressed as means ± standard error of the mean (SEM). Some data were normalized as a percentage of the control values (100%). One-way ANOVA and one-way ANOVA repeated measures followed by post hoc test (Bonferroni) were used for statistical comparisons and P < 0.05 was considered to be statistically significant. A Kolmogorov-Smirnov test was used to evaluate statistical significance for cumulative data.

All experiments were approved by the local Institutional Animal Care and Use Committee and were consistent with the guidelines of the International Association for the Study of Pain, the National Institutes of Health Guide for the Care and Use of Laboratory Animals and the European Communities Council Directive of 24 November 1986 (86/609/EEC).

## Results

Spinal cord slices were incubated in 60 nM TNFα for at least 2 h before the actual experiments. Recordings from 16 neurons in slices incubated with TNFα were made altogether. The application of the endogenous TRPV1 receptor agonist OLDA (0.2 μM) increased mEPSC frequency in 10 of the 16 DH neurons tested. At the end of each recording, 0.2 μM capsaicin was administered to confirm input from nociceptive primary afferents expressing TRPV1 receptors. Capsaicin is known to increase mEPSC frequency in superficial DH neurons in a concentration-dependent manner via presynaptic TRPV1 receptors localized on central terminals of DRG neurons [28,32]. Capsaicin application in our experiments increased mEPSC frequency in 13 of the 16 DH neurons by 372.6 ± 78.1%. All 10 neurons that responded to the OLDA application were also responsive to the capsaicin application (10 out of 13).

**Figure 1:**
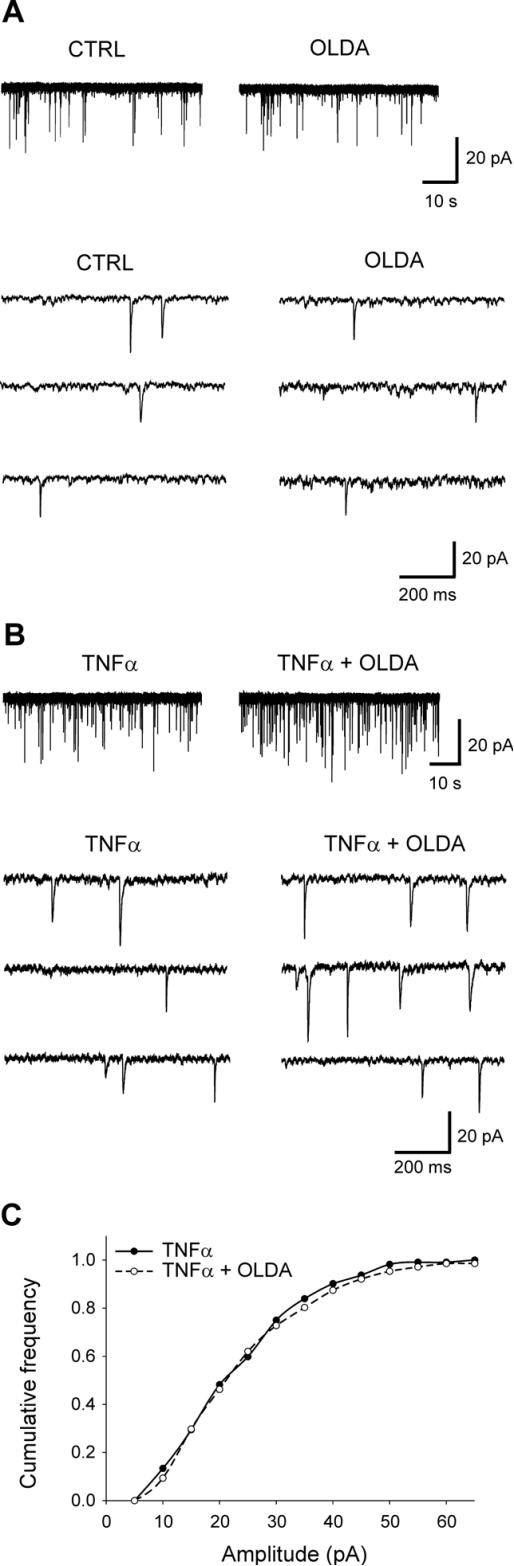
**Application of a low concentration of OLDA (0.2 μM) did not change mEPSC frequency in a superficial dorsal horn neuron, as recorded under control conditions (**A**), while a significant increase in mEPSC frequency was observed in slices incubated with the cytokine TNFα (60 nM; B)**. The amplitude of the mEPSCs was not changed after OLDA application in this neuron (**C**).

**Figure 2:**
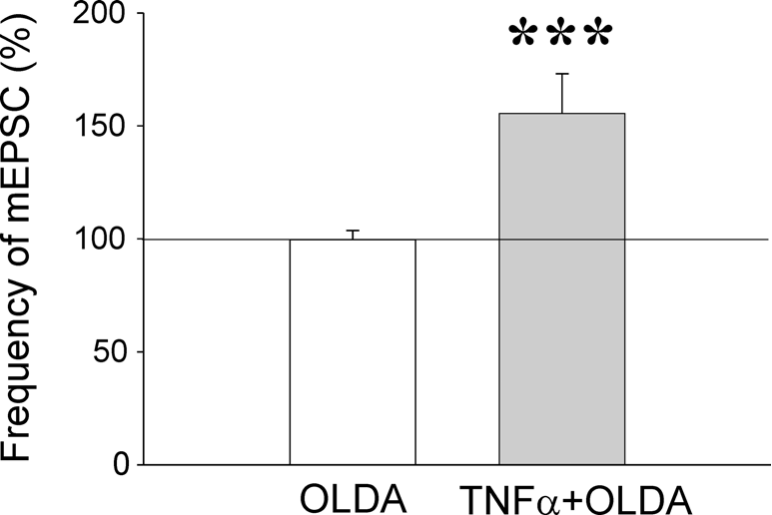
**Application of a low concentration of OLDA solution (0.2 μM) did not change mEPSC frequency in neurons, as recorded under control conditions (n = 25)**. The same OLDA application significantly increased mEPSC frequency in neurons recorded in spinal cord slices incubated with TNFα (60 nM; n = 10; ***P < 0.001). Values are given in percent of the corresponding mEPSC frequency before the OLDA application (100%).

**Figure 3:**
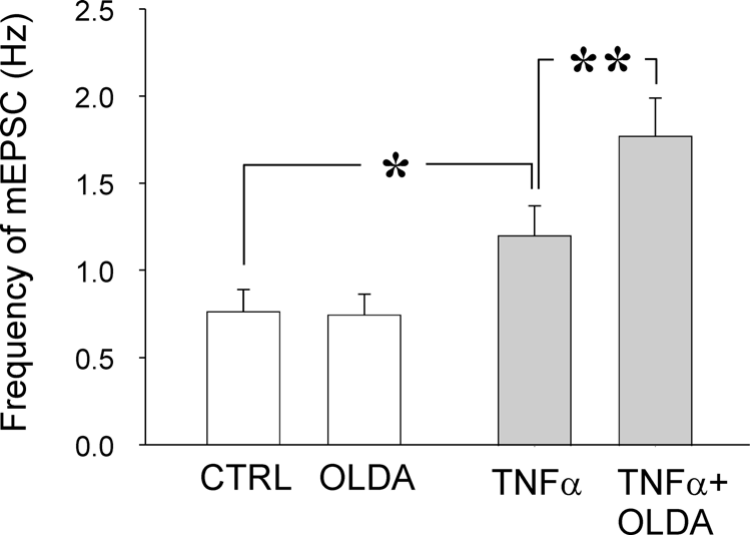
**In control slices without TNFα pretreatment, OLDA application did not induce a significant change in mEPSC frequency**. Incubation with the cytokine TNFα increased basal mEPSC frequency (n = 10; *P < 0.05) in a population of DH neurons with capsaicin-sensitive primary afferent fibre inputs, when compared to recordings from control slices without TNFα incubation (n = 25). The high statistical significance of the mEPSC frequency increase during OLDA application in neurons after TNFα incubation was also evident without data normalization (**P < 0.01).

We have shown previously that application of a low concentration of OLDA (0.2 μM) does not evoke any changes under control conditions [28]. Basal mEPSC frequency in the control recordings established as 100% did not change during OLDA (0.2 μM) application (99.6 ± 4.0%; n = 25; Figs. 1A and 2). In the actual measurements, the low concentration of OLDA solution (0.2 μM) applied under the same experimental conditions increased the mEPSC frequency to 155.5 ± 17.5% (P < 0.001; n = 10) in acute spinal cord slices incubated with TNFα, when compared to the control values before the OLDA application (Figs. 1B and 2). Capsaicin application in this population of neurons increased mEPSC frequency to a higher level (387.4 ± 109.5%; n = 10) than did the endogenous agonist. Increase in the absolute mEPSC frequency during OLDA application in neurons incubated with TNFα (1.78 ± 0.22 Hz) was also statistically significantly different from the control mEPSC frequency before OLDA application (1.21 ± 0.17 Hz; P < 0.01; Fig. 3) and from the basal mEPSC frequency recorded in control slices without the TNFα pretreatment (0.76 ± 0.13 Hz; n = 25; P < 0.001). The basal mEPSC frequency (1.21 ± 0.17 Hz) recorded in neurons in TNFα incubated slices was significantly higher (P < 0.05) when compared to the basal mEPSC frequency (0.76 ± 0.13 Hz) recorded from neurons in control slices (Fig. 3).

Incubation of spinal cord slices with TNFα did not change the average mEPSC amplitude in the recorded neurons during OLDA (23.02 ± 2.98 pA; n = 10) application compared to control values before its application (21.98 ± 2.50 pA) and compared to mEPSC amplitude in neurons from control slices (24.12 ± 1.23 pA; n = 25). This is documented also by a cumulative amplitude distribution (Fig. 1C).

All recorded superficial DH neurons with input from TRPV1 receptors containing primary nociceptive fibres incubated with TNFα had a significantly higher basal frequency of mEPSCs (1.35 ± 0.20 Hz; n = 13; P < 0.01) compared to the same population of control neurons (0.76 ± 0.08 Hz; n = 53) [28]. In contrast, neurons without input from primary afferents containing TRPV1 receptors and incubated with TNFα had a lower basal mEPSC frequency (1.02 ± 0.56 Hz; n = 3) compared to the same population of neurons in control slices (2.07 ± 0.39 Hz; n = 23; P > 0.05). Average basal mEPSC frequency of all of the recorded neurons was not different between the neurons recorded in slices incubated with TNFα (1.28 ± 0.18; n = 16) and in control slices (1.13 ± 0.14; n = 76). While the number of recorded neurons without presynaptic input from capsaicin sensitive fibres was very low (n = 3), we cannot deduce from our results if there was any significant effect of TNFα on the mEPSC frequency in this population of neurons. There was no change in average mEPSC amplitude in DH neurons with input from capsaicin sensitive and insensitive primary afferent fibres incubated with TNFα compared to the appropriate populations of neurons in control slices without TNFα incubation.

## Discussion

Application of a low concentration (0.2 μM) of the TRPV1 endogenous agonist OLDA does not evoke any changes in mEPSC frequency recorded from superficial dorsal horn neurons under control conditions without TNFα incubation [28]. In the present experiments we have demonstrated that TNFα pretreatment significantly increases sensitivity to OLDA application in 10 (~60%) out of the total of 16 recorded neurons. Our hypothesis assumes that this effect is mediated by TNFR-induced modulation of TRPV1 receptors. It thus seems plausible to suggest that both TRPV1 and TNFR receptors have to be expressed in the presynaptic endings for this effect to be present. Out of the 16 neurons, 3 did not respond to the capsaicin application, implying a lack of TRPV1 receptors in these neurons. In the population of 13 neurons responding with increased mEPSC frequency to the capsaicin application, 3 neurons did not respond to the OLDA application, possibly due to an absence of TNFR receptors, while 10 out of the 13 neurons (~77%) responded to the OLDA application.

The importance of TNFR1 receptors for development of neuropathic pain syndromes appears to be well documented. TNFR1 receptors are not only present on DRG neuronal bodies but also at numerous central branches of DRG neurons in the spinal cord dorsal horn [33]. A crucial role of TNFR1 receptor activation in the experimental model of neuropathic pain using chronic constriction injury of the sciatic nerve has been demonstrated using neutralizing antibodies to the TNFR1 receptor to reduce thermal hyperalgesia and mechanical allodynia [34]. Ventral root transection accompanied with mechanical allodynia and thermal hyperalgesia increases immunoreactivity of TNFα and TNFR1 receptors in ipsilateral DRG and bilaterally in the spinal cord DH [35]. A role for TNFR2 receptors in heat hyperalgesia has been implicated in a model of cancer pain where TRPV1 protein up-regulation in DRG neurons is attenuated in TNFR2 knock-out mice [4].

Coexpression of mRNA for TNFR1 and TRPV1 receptors [17] and colocalization of TNFR1 and TRPV1 immunoreactivity [29] in DRG neurons has been reported. Long-term application of TNFα (48 h) increases the number of TRPV1 immunopositive DRG neurons in culture. This increased expression of TRPV1 receptors is dependent on ERK (extracellular regulated kinase = p44/p42 MAP kinase) activation and TNFR1 receptors, as has been shown using DRG neurons isolated from TNFR1^-/- ^knock out mice [29]. In contrast, TRPV1 receptor up-regulation in DRG neurons is dependent on TNFR2 receptors in tumor-bearing mice [4]. The increase of TRPV1 protein level after TNFα application correlates well with a previous demonstration of an increased number of DRG neurons labeled with cobalt staining after TRPV1 receptor activation by capsaicin in neurons cultivated with TNFα [31]. The increased cobalt staining in these experiments is dependent on the activation of cyclooxygenase, in contrast to other findings [29]. The change in capsaicin sensitivity after TNFα treatment is rapid, with an increase after 4 h of incubation [31]. TNFα stimulation of cultured DRG neurons produces an increase in TRPV1 receptor protein level after 1 and 2 h of incubation [4]. In trigeminal neurons, TNFα application increases CGRP release after capsaicin stimulation [30]. In these experiments, doubling of TRPV1 receptor mRNA was evident only after 30 min of TNFα incubation.

In our experiments the cell bodies of DRG neurons were absent during the TNFα incubation of spinal cord slices. The increased sensitivity to OLDA application was thus most likely mediated through TRPV1 receptors phosphorylation and/or their translocation from the cytoplasm to the presynaptic ending membrane. Increased sensitivity of TRPV1 receptors to agonists due to PKC- or PKA-mediated phosphorylation has been repeatedly demonstrated [36]. Also, TNFα incubation (5 min) has been shown to have a fast potentiating effect on capsaicin-stimulated increases in intracellular accumulation of Ca^2+ ^ions [30]. Capsaicin-evoked currents are robustly potentiated after only 60 s of TNFα application in cultured DRG neurons, and this effect is mediated via activation of PKC or p38/MAP kinase [4]. It is thus probable that the effect of TNFα on TRPV1 receptors is mediated by similar pathways in our experiments, while transcriptional changes may also play an important role under pathological conditions *in vivo*, where spinal TNFα has been shown to play an important role [37].

In our experiments we did not record any significant change in the mEPSC amplitude, which is in good agreement with previous findings [38,39]. However, postsynaptic changes due to TNFα incubation could have most likely also occurred. Regulation of NMDA receptor activity or AMPA receptor expression by TNFα has been described for other areas of the CNS [40]. Neutralization of TNFα in the rostral ventromedial medulla (RVM), a major component of brainstem descending pain modulatory circuitry, attenuates phosphorylation of NMDA receptor NR1 subunits [41]. TNFα induces enhancement of synaptic efficacy by increasing surface AMPA receptor expression in hippocampal neurons [42]. It has also been documented that TNFα enhances spontaneous EPSCs and potentiates AMPA- and NMDA-mediated currents due to up-regulation of spinal chemokine MCP-1 and activation of its postsynaptic CCR2 receptors in DH neurons [43]. Acute TNFα application in spinal cord slices increases the frequency of spontaneous EPSCs in some lamina II neurons (8 of 14 neurons), and also increases AMPA- (5 of 9 neurons) and NMDA- (all recorded neurons) stimulated currents [38]. Increase of sEPSC and mEPSC frequencies during acute application of TNFα has been confirmed by Youn et al. (2008) [39], together with the observation of a TNFα-stimulated increase in spontaneous IPSC frequency. From previously published results and our study, it is evident that TNFα- induced modulation of excitatory synaptic transmission in the spinal cord dorsal horn is a complex process mediated by fast mechanisms and by slow transcription-dependent changes.

Our results suggest that increased levels of TNFα and its receptors under pathological conditions may have considerable impact on the function of spinal cord TRPV1 receptors. The resulting increased sensitivity of TRPV1 receptors to endogenous agonist may significantly modulate nociceptive synaptic transmission in the spinal cord dorsal horn. While additional experiments are needed to elucidate the specific mechanisms involved in TNFα-induced modulation of TRPV1 receptor function, it is clear that these results may also point to new targets for pain therapy.

## Competing interests

The authors declare that they have no competing interests.

## Authors' contributions

JP conceived and designed the study, DS performed and analyzed the experiments. DS and JP drafted the manuscript. Both authors have read and approved the final version of the manuscript.
